# Non-destructive methods to assess health of wild tropical frogs (túngara frogs: *Engystomops pustulosus*) in Trinidad reveal negative impacts of agricultural land

**DOI:** 10.1007/s11356-022-20105-4

**Published:** 2022-04-24

**Authors:** Frances Orton, Stephanie Mangan, Laura Newton, Alexis Marianes

**Affiliations:** 1 School of Health and Life Sciences, University of the West of Scotland, Paisley, PA1 2BB Scotland; 2 School of Science, University of Waikato, Hamilton, 3216 New Zealand; 3 Sustainable Innovation Initiatives, 9735 SW 166 Terr, Miami, FL 33157 USA

**Keywords:** Amphibian, Freshwater, Pollution, Pesticides, Ecotoxicology, Endocrine disruption, Reproduction, Biodiversity

## Abstract

Amphibians are threatened globally with at least 43% of species declining and the most important stressor being habitat loss or degradation. Amphibians inhabiting highly biodiverse tropical regions are disproportionately threatened; however, the effects of landscape alterations on amphibian health are virtually unknown. In this study, we utilised non-destructive techniques to compare size (weight, snout-vent length [SVL]), body condition, male secondary sexual features (forelimb width, nuptial pad length) and breeding success (egg number, fertility [percentage fertilised eggs], hatching success) in túngara frogs (*Engystomops pustulosus*) collected from reference (*n* = 5), suburban (*n* = 6) and agricultural (*n* = 4) sites in Trinidad; characterised by presence/absence of crops/houses. All measured endpoints were negatively impacted in frogs collected from agricultural sites. The largest effect was observed for hatching success (2.77-fold lower) and egg number (2.5-fold lower). Less pronounced effects were observed on male frogs (weight: 1.77-fold lower; SVL: 1.18-fold lower; forelimb width: 1.33-fold lower; nuptial pad length: 1.15-fold lower). Our findings demonstrate negative impacts of agricultural sites on túngara frog health, with the number of viable offspring reduced by almost one third. The methods outlined here are technically simple and low-cost and thereby have potential for application to other species in order to investigate the potential impacts of habitat degradation on amphibian health. Furthermore, as these methods are non-destructive, they could be used to investigate the potential contribution of frog size and/or reproductive capability as a causative factor contributing to population declines in threatened species, which is particularly pressing in tropical regions.

**Supplementary Information:**

The online version contains supplementary material available at 10.1007/s11356-022-20105-4.

## Introduction

Amphibians are the most threatened vertebrate group, with at least 43% of species declining (Díaz et al. 2019). Several anthropogenic drivers are indicated in causing declines, including habitat loss and degradation, pollution, disease, climate change and invasive species. Habitat loss or degradation and pollution are listed as the first and second most important causes of amphibian declines globally, However, these stressors often co-occur as agricultural intensification and urbanisation cause both habitat alterations, as well as pollutant discharges (Díaz et al. 2019). Tropical regions encompass highly biodiverse amphibian assemblages and are disproportionately impacted by drivers of decline (Hof et al. 2011); however, little is known regarding the effects of habitat loss, habitat degradation or pollution, on tropical amphibians. Apart from mortality caused by chytridiomycosis (e.g. Latin America: Lips et al. 2008), the impacts of other stressors—for example habitat loss/alteration and pollution—on the health of tropical amphibian species are poorly defined (Ghose et al. 2014). To date, only a handful of studies have investigated amphibian biodiversity differences between degraded (agricultural) versus ‘reference’ (non-agricultural) areas, with all reporting lower biodiversity in the agricultural areas (Mexico: Lips et al. 2004; Guatemala: Mendelson et al. 2004; Brazil: Ferrante and Fearnside 2020; Sri Lanka: Rajakaruna et al. 2007; India: Rathod and Rathod 2013). Investigation into intraspecies differences between polluted and non-polluted sites have also been occasionally reported, for example lower acetylcholinesterase activity and genotoxicity in tadpoles (Brazil: Santos et al. 2015; Gonçalves et al. 2019) and in adult frogs (Hegde and Krishnamurthy 2014; Nigeria: Taiwo et al. 2014; India: Hegde et al. 2019) was reported in individuals collected from polluted environments. Additionally, there are reports of undersized frogs from agricultural versus reference environments in India (Hegde and Krishnamurthy 2014) and Thailand (Thammachoti et al. 2012).

There is a greater depth of research into health of wild amphibians from temperate regions, and these have indicated a range of effects on morphology of individuals collected from degraded/polluted versus reference/unpolluted environments. For example, the toads *Spea multiplicata*, *Spea bombifrons* and *Bufo cognatus* were smaller in cultivated versus uncultivated wetlands in the USA (Gray and Smith 2005), and common toads (*Bufo bufo*) were smaller in an agricultural versus a non-agricultural site in the UK (Orton et al. 2014), although natterjack toads (*Epidalea calamita*) were reported to be larger from agricultural sites (Zamora-Camacho and Comas 2017). Less commonly, secondary sexual features in males, such as nuptial pad size and/or number, and forelimb width, have been reported to differ in wild amphibians collected from polluted versus reference populations, and both smaller (McCoy et al. 2008; Orton et al. 2014) and larger (Zamora-Camacho and Comas 2017) size has been reported. These features have been shown to have importance to breeding success in laboratory exposed (*Silurana tropicalis*: Orton et al. 2020) and as well as in wild (*Rana luteiventris*: Greene and Funk 2009) frogs. In addition, toadspawn from *Bufo bufo* (Orton and Routledge 2011; Bókony et al. 2018) or *Anaxyrus terrestris* (Metts et al. 2013) collected from polluted environments (agricultural or coal combustion (metal) contamination) and reared in laboratory ‘clean’ water displayed lower hatching success (Orton and Routledge 2011; Metts et al. 2013) or reduced offspring fitness, characterised by reduced development rates and lower body mass (Bókony et al. 2018). To the authors’ knowledge, neither breeding success (egg number, fertility, hatching success) nor male secondary sexual characteristics (forelimb width, nuptial pad) have been investigated in wild tropical frogs to date.

In this study, we set out to investigate the impacts of agricultural and suburban sites on body size, male secondary sexual characteristics and reproductive success in túngara frogs (*Engystomops pustulosus*) utilising non-destructive sampling methods. Túngara frogs are leptodactylid frogs (Duellman and Trueb 1994) that are widely distributed across Latin America and the Caribbean (Weigt et al. 2005). They are found in small water bodies where amplectant pairs lay eggs in foam nests (Heyer and Rand 1977), which typically hatch in ~ 48 h (Duellman and Trueb 1994). Túngara frogs are prolonged breeders and exhibit female mate choice resulting in individual males and females forming amplectant pairs (Ryan 1985). They are a well-studied species (e.g. Ryan 1983, 1985, 2010), facilitating comparisons of morphological and reproductive endpoints in new study populations.

## Methods

### Site characteristics

**Fig. 1:**
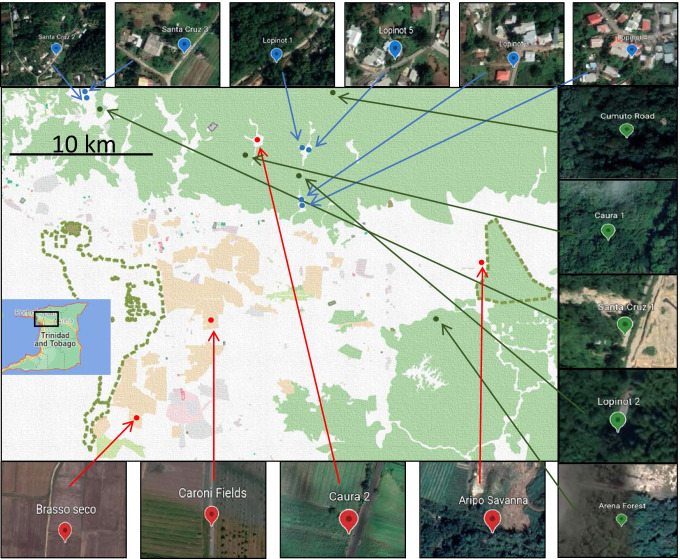
Map showing site locations and land use (white = buildings, brown = farming land, green = forested, pink/grey = large settlement). Green points = reference sites (2016: Cumuto road (R.CR), Caura 1 (R.C1), Arena Building (R.AB), Lopinot 2 (R.L2); 2018: Santa Cruz 1 (R.SC1)). Blue points = suburban sites (2016: Lopinot 3, 4 (U.L3, U.L4), Lopinot 1 (U.L1); 2018: U.L1, Lopinot 5 (U.L5), Santa Cruz 2, 3 (U.SC2, U.SC3)). Red points = agricultural sites (2016: Brasso Seco (A.BS), Aripo Savanna (A.AS), Caura 2 (A.C2); 2018: Caroni Fields (A.CF))

Sites were defined as individual ponds and selection was based on the existence of a túngara frog population, practical considerations (no more than a 2-h drive from laboratory, safety, access), as well as attempting to select approximately equal numbers of each site type (see Fig. 1 for map of sites). As a first step in site characterisation, visual inspection was used (i.e. suburban—ponds in hamlets or outskirts of towns [< 50 m from nearest dwelling]; agricultural—ponds in close proximity to crop fields [< 50 m from nearest crop field]; reference—no houses or crops in close proximity to the pond [> 200 m distance]). Additionally, in 2019, Google Maps was used to investigate whether initial visual site designation tallied with satellite images. For each site, the format of GPS coordinates were converted for compatibility with Google Maps (https://www.gps-coordinates.net/gps-coordinates-converter) and a satellite photo was used to characterise the surrounding environment (presence/absence of crops and houses). All sites were deemed independent, with the two closest sites 559 m apart from each other with a road and/or forest separating the two sites (Santa Cruz 2 and Santa Cruz 3). No differences between initial visual characterisation and that using mapping were observed (see Supplemental Table S1 for site coordinates and Supplemental Figure S1 for individual site images).

Since car access was required to carry equipment and samples, all of the sites, including the reference sites, were situated in close proximity to roads (range: < 1–20 m) with varying amounts of traffic. Therefore, the term ‘reference’ is not synonymous with ‘undisturbed’. Data were collected from 15 sites (one site was visited in 2016 and 2018), comprising reference (5 sites: 2016—Cumuto road (R.CR), Caura 1 (R.C1), Arena Building (R.AB), Lopinot 2 (R.L2); 2018—Santa Cruz 1 (R.SC1)), suburban (6 sites: 2016—Lopinot 3 (U.L3), Lopinot 4 (U.L4), Lopinot 1 (U.L1); 2018—U.L1, Lopinot 5 (U.L5), Santa Cruz 2 (U.SC2), Santa Cruz 3 (U.SC3)) and agricultural (4 sites: 2016—Brasso Seco (A.BS), Aripo Savanna (A.AS), Caura 2 (A.C2); 2018—Caroni Fields (A.CF)).

### Experimental design

Sampling took place during the rainy season and amplectant pairs of frogs were collected in both years on warm nights when breeding activity was high (June–October/November). On arrival at each sampling site (between 4:00 and 5:00 pm), water quality (temperature, pH, dissolved oxygen [DO], salinity, conductivity, total dissolved solids [TDS]—Hach multimeter) and nitrogenous compounds to assess potential agricultural inputs (nitrate, ammonia—API test kit) basic habitat features (pond description, surrounding vegetation, proximity to houses, crops) and location (GPS) were recorded. All amplectant pairs of túngara frogs that were observed were then collected. Upon collection, each pair was placed in a plastic tub filled with site pond water. Amplectant pairs were transported to the laboratory (University of the West Indies) to deposit foam nests overnight and kept at ambient temperature (i.e. equivalent to outside air temperature, not recorded). The next day, all pairs had separated, and adult males and females were photographed, weighed and snout-vent length (SVL) measured. Forelimb width (FLW) of males was measured using photographs of the forelimb (2016/2018: Adobe Photoshop, see Supplemental Text S1). In 2016, nuptial pad length in males was measured with callipers and in 2018 nuptial pad length was measured from photos (Adobe Photoshop, different methods were used due to an unforeseen error in the data collection: see Supplemental Text S1). Nests were relocated to new tanks containing natal pond water and placed over a white background to facilitate photographs of hatched tadpoles. The foam nests were monitored until hatching of tadpoles (24–48 h after collection). All adults and tadpoles were returned to their natal ponds after breeding status metrics had been recorded, and within 60 h of collection.

### Breeding success

After hatching of tadpoles had occurred (24–48 h after collection), the foam nest was removed and gently shaken before being dissected to release any remaining tadpoles. Tanks containing tadpoles were then photographed and the number of tadpoles determined using the cell count function of ImageJ (National Institutes of Health, Bethesda, MD). Comparison of visual and ImageJ tadpole counting was carried out with 10 randomly selected tank photos, with an *R*^2^ = 0.96, indicating high similarity between the two methods. Hatching success was determined by counting the number of hatched tadpoles. The number of unhatched eggs was also counted, and egg number (total number of eggs) was calculated by adding together the number of unhatched eggs and the number of hatched tadpoles. Fertility (percentage fertilised) was calculated by dividing the number of hatched tadpoles by egg number (× 100 for percentage).

### Chemical analysis

Analysis of agricultural chemicals was undertaken utilising destructive methods. This was justified from an ethical perspective as typically, few tadpoles survive the larval period to complete metamorphosis (e.g. 5%: Calef 1973), and therefore, sacrificing a small proportion of anurans from their larval life stage is unlikely to impact populations. For 5 out of the 10 sites surveyed in 2016 (R.C1 and R.AB (reference), U.L1 and U.L4 (suburban), A.AS (agricultural)), chemical analysis of a pooled sample of whole wild caught tadpoles was undertaken (283 chemicals, detection limits: 0.01 mg/kg). Due to financial constraints, the analysis of chemicals in was not possible in 2018. From each site sampled in 2016, 30 to 50 g of wild caught tadpoles (~ 50–100 tadpoles per site) were collected using a net, with the total sample placed in a 50-ml falcon tube. Tadpole samples were collected approximately 2 weeks after adult pairs had been caught for measurement of the other endpoints. The falcon tubes containing each tadpole sample were immediately placed on dry ice on site, stored in − 80 °C freezer and transported to Almeria, Spain (Laboratorio Analitico Bioclinico) for chemical analysis using gas chromatography–mass spectrometry or liquid chromatography-tandem mass spectroscopy (depending on the analyte). Sampling of biota, instead of water sampling, was chosen in order to detect only the bioavailable fraction of chemicals. In addition, sampling of tadpoles reflected ‘early-life exposure’, which is well known to be critically important to long-term health of vertebrate organisms (e.g. Coe et al. 2010). All analytical methods were optimised for chemicals testing in the tadpole matrix. See Supplemental Text S2 for more details of the analytical methods and Supplemental Tables S2 and S3 for the list of chemicals analysed.

### Statistical analysis

Some data were removed prior to analysis: in one case a female escaped (reference: weight and SVL not measured), in another case the female SVL was not measured in error (suburban) and for 12 pairs of frogs, no nest was made (5 reference, 5 suburban, 2 agricultural). The condition of individuals was calculated using a ratio-based condition index (CI: body weight/SVL), which has been shown to be effective for comparing condition between amphibian populations (Labocha et al. 2013). Data ranges for all endpoints in 2016 versus 2018 were compared, and as they showed a high degree of overlap (Table S3), data were pooled in order to maximise sample size. Pooled data were then tested for normality (Kolmogorov–Smirnov test). For data that were not normally distributed (all endpoints except for CI and FLW), analyses for differences between site types were conducted using a Kruskal–Wallis test, followed by a Dunn’s post hoc test (reference compared to suburban or agricultural, multiplicity adjusted *p* value is reported). For the forelimb width data, a one-way ANOVA was conducted, followed by a Holm-Sidak post hoc test (Bonferroni multiplicity adjusted *p* value is reported). Additionally, in order to identify if observed differences between sites were confounded by the size of frogs, analyses of covariance (using non-linear regression) were conducted on endpoints where confounding effects may be expected according to the published literature. For females, differences in egg number between sites were analysed with female weight as the covariate (Poisson fit (appropriate for count data), followed by a likelihood ratio test) as these measurements are sometimes reported to be correlated (e.g. Prado and Haddard, 2005). For males, differences in forelimb width/nuptial pad length between sites were analysed with male weight as the covariate (least squares fit, followed by an extra sum of squares *F* test), as overall male size could influence FLW or nuptial pad length (e.g. Orton et al. 2020). Apart from temperature and nitrate (pooled between years, not normally distributed), differences in water quality between site types were not analysed statistically due to low sample size. All analyses were carried out using GraphPad Prism. *p* values of < 0.05 were deemed significant.

## Results

### Field sampling

In total, data were collected from 15 sites (reference: 5 sites—R.CR, R.C1, R.AB, R.L2, R.SC1; suburban: 6 sites—U.L3, U.L4, U.L1, U.L5, U.SC2, U.SC3; agricultural: 4 sites—A.BS, A.AS, A.C2, A.CF), with site U.L1 visited in both years; and water bodies comprised either puddles or drainage ditches (Table 1). Conductivity, salinity and TDS levels were higher at suburban and agricultural sites, compared to the reference site where these measurements were taken, whereas pH values were lower (not analysed statistically, Table 1). Temperature and nitrate levels did not differ between site categories (Kruskal–Wallis, *p* = 0.15; Table 1).

**Table 1:** Characteristics of field sites

Site	Year	Temp	pH	DO	Sal	Cond	TDS	Nitr	NH_3_	Description
R.CR	2016	24.5						5		1 m^2^ puddle in drainage ditch in side of road. Short grassland on slope above
R.C1	2016	23.8						5		2 × 3 m puddle next to abandoned house. Short grassland, 10 m above river
R.AB	2016	24.7						10		Several puddles from heavy machinery tracks in forest opening. Grassland/edge of forest
R.L2	2016	24.6						0		Along ditch in roadside, ~ 3 m long, narrow puddle. Steep cliffs either side of road, short grass around puddle
R.SC1	2018	23.45	10.3	2.38	0.06	123	88	2.5	2.0	Long drainage ditch, 30 m along side of road. Grassland/edge of forest
Reference sites		24.21^a^	10.3	2.38	0.06	123	88	4.5^a^	2.0	
U.L3	2016	24.5						5		0.5 m^2^ puddles in roadside drainage ditch. Short grass nearby
U.L4	2016	23.9						0		Drainage ditch on roadside plus large grass area with 2 m^2^ puddle. Tall grasses
U.L1	2016	24.3						15		4 × 2 m puddle on small road, next to construction. Tall grasses and trees behind
U.L1	2018	24.2	8.67	3.26	0.06	126	87	0.5	0.75	4 × 2 m puddle on small road, next to construction. Tall grasses and trees behind
U.L5	2018	25.1	9.9	2.49	0.13	280	184	2.5	0.5	Several 0.5 m^2^ puddles within a small residential area. Tall grasses nearby
U.SC2	2018	24.9	7.2	1.86	0.12	261	170	0	2.0	2 × 1 m puddle within 5 m of two residential dwellings. Short grass nearby
U.SC3	2018	24.2	6.16	1.32	0.19	405	264	0	0.5	2 × 1 m puddle within 5 m of a residential dwellings. Short grass nearby. Trees nearby
Urban sites		24.4^a^	7.98^a^	2.23^a^	0.13^a^	268^a^	176^a^	3.29^a^	0.94^a^	
A.BS	2016	24.1						0		0.5 m long puddle in shallow dip in short grassland. Crop fields and hedge behind
A.AS	2016	24.5						0		2 × 1 m puddle, off main road on track. Grassland going into forest, crop fields observed within 5 m
A.C2	2016	25.0						0		4 × 1.5 m puddle at the end of a driveway on roadside. At the bottom of a crop fields
A.CF	2018	23.9	5.7	3.36	0.14	190	123	1	2.5	1 m long drainage ditch beside the road. Crop fields and hedge behind
Agricultural sites		24.4^a^	5.7	3.36	0.14	190	123	0.25^a^	2.5	

^a^Mean values. Abbreviations: *R.CR*, Cumuto road; *R.C1*, Caura 1; *R.AB*, Arena Building; *R.L2*, Lopinot 2; *R.SC1*, Santa Cruz 1; *U.L3*, Lopinot 3; *U.L4*, Lopinot 4; *U.L1*, Lopinot 1; *U.L5*, Lopinot 5; *U.SC2*, Santa Cruz 2; *U.SC3*, Santa Cruz 3; *A.BS*, Brasso Seco; *A.AS*, Aripo Savannah; *A.C2*, Caura 2; *A.CF*, Caroni Fields; *Sal.*, salinity; *Cond.*, conductivity; *Nitr.*, nitrate. See Supplemental Table S1 for map of sites and coordinates

In total, 205 frog pairs were collected (2016—63 pairs; 2018—142 pairs, Table 2) from 15 sites (reference = 5, suburban = 6, agricultural = 4, Table 2, Fig. 1); 9 of these sites were visited in 2016 and 5 sites in 2018, with one site visited in both years (U.L1—Lopinot 1). Fewer pairs overall were collected in 2016 (63 out of 205; 31%) versus 2018 (142 out of 205; 69%). The proportion of pairs sampled was broadly similar between different site categories in both 2016 (reference = 27%, suburban = 43%, agricultural = 30%) and in 2018 (reference = 37%, suburban = 39%, agricultural = 25%), as were the total number of amplecting pairs collected in the different site categories (reference = 69; suburban = 82; agricultural = 54).

**Table 2:** Summary of sampling effort

Site	Year	Type	Month	No. visits	Pairs
R.CR	2016	Ref	Jun	1	2
R.C1	2016	Ref	Jun	1	1
R.AB	2016	Ref	Jun–Jul	3	8
R.L2	2016	Ref	Jun	2	6
Summary 2016			Jun–Jul	7	17
R.SC1	2018	Ref	Aug–Oct	5	52
Summary 2018			Aug–Oct	5	52
Reference summary			Jun–Oct	12	69
U.L3	2016	Urb	Jun–Jul	2	7
U.L4	2016	Urb	Jun–Jul	4	11
U.L1	2016	Urb	Jun–Jul	3	9
Summary 2016			Jun–Jul	9	27
U.L1	2018	Urb	Jul	2	21
U.L5	2018	Urb	Jul	1	1
U.SC2	2018	Urb	Aug–Nov	3	5
U.SC3	2018	Urb	Aug–Nov	4	28
Summary 2018			Jul–Nov	10	55
Suburban summary			Jun–Nov	19	82
A.BS	2016	Agr	Jun–Jul	2	3
A.AB	2016	Agr	Jun–Jul	5	15
C.C2	2016	Agr	Jun	1	1
Summary 2016			Jun–Jul	8	19
A.CF	2018	Agr	Oct	4	35
Summary 2018			Oct	4	35
Agricultural summary			Jun–Oct	12	54
Complete sampling effort			Jun–Nov	43	205

Abbreviations: *Ref*, reference; *Urb*, suburban; *Agr*, agricultural; *R.CR*, Cumuto road; *R.C1*, Caura 1; *R.AB*, Arena Building; *R.L2*, Lopinot 2; *R.SC1*, Santa Cruz 1; *U.L3*, Lopinot 3; *U.L4*, Lopinot 4; *U.L1*, Lopinot 1; *U.L5*, Lopinot 5; *U.SC2*, Santa Cruz 2; *U.SC3*, Santa Cruz 3; *A.BS*, Brasso Seco; *A.AS*, Aripo Savannah; *A.C2*, Caura 2; *A.CF*, Caroni Fields

### Morphology and breeding success

**Fig. 2:**
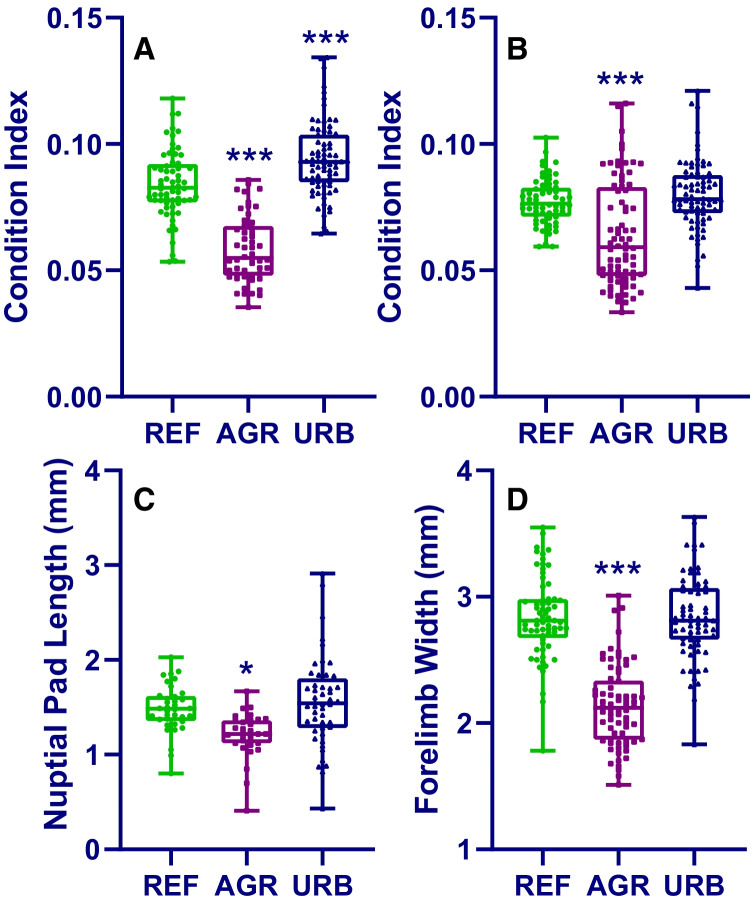
Condition index (females: **A**; males: **B**), nuptial pad length (**C**) and forelimb width (**D**) in túngara frogs collected from reference (REF), agricultural (AGR) and suburban (URB) sites. Box and whisker plots, showing data range (whiskers) and all data values, line represents median value. Statistical significance asterisks are for suburban or agricultural site types, compared to reference site types (*p < 0.05, **p < 0.01, ***p < 0.001)

**Fig. 3:**
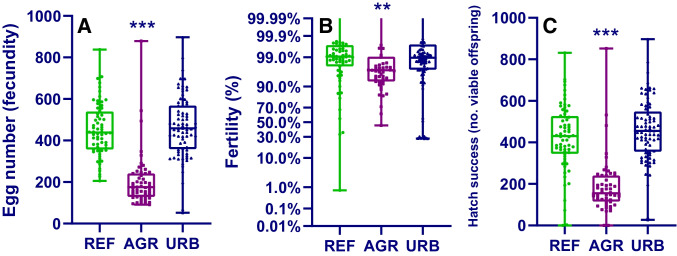
Egg number (**A**), fertility (**B**: probability scale on *y* axis) and hatching success (**C**) in pairs of túngara frogs collected from reference (REF), agricultural (AGR) and suburban (URB) sites. Box and whisker plots, showing data range (whiskers) and all data values, line represents median value. Statistical significance asterisks are for suburban or agricultural site types, compared to reference site types (*p < 0.05, **p < 0.01, ***p < 0.001)

Few differences were observed between frogs collected from reference or suburban site types, with the only significant differences being larger females collected from suburban sites (female weight: 1.15-fold higher; female CI: 1.13-fold higher: Fig. 2, 3 and Table S4). In contrast, all measured endpoints were significantly reduced in frogs collected from agricultural site types compared to those collected from reference site types (Figs. 2, 3 and Table S4, Kruskal–Wallis/ANOVA, Dunn’s/Holm-Sidak, *p* < 0.026). For morphological characteristics, the magnitude of difference between frogs collected from reference versus agricultural site types ranged from 1.15-fold lower for nuptial pad length to 2.16-fold lower for female weight (Table S4). For female frog morphology, effects ranged from 1.3-fold lower (CI) to 2.16-fold lower (weight), and for male frog morphology, effects ranged from 1.15-fold lower (nuptial pad length) to 1.77-fold lower (weight). For breeding success, the magnitude of difference between frogs collected from reference versus agricultural site types ranged from 1.06-fold lower (fertility) to 2.77-fold lower (hatching success). Therefore, effects were the most pronounced for breeding success, in particular, the number of hatched tadpoles (2.77-fold lower) and fecundity (2.5-fold lower), compared to morphological endpoints (Table S4).

### Analyses of covariance

Analyses of egg number with female weight as the covariate showed significant differences between site types (semi-log, Poisson fit, likelihood ratio test *p* < 0.0001), indicating that differences in fecundity between site types occurred independently of female size. Analyses of forelimb width and nuptial pad length with male weight as the covariate showed significant differences between site types for forelimb width (linear, least squares fit, extra sum of squares *F* test *p* < 0.0001), indicating that this effect occurred independently of male size; but not for nuptial pad length (linear, least squares fit, extra sum of squares *F* test *p* = 0.395), indicating that differences between site types for this endpoints were not independent of male weight (for graphical representation of correlations see Supplemental Figure S2).

### Chemistry

None of the chemicals analysed in tadpole tissue was detected from any of the sampling sites.

## Discussion

The aim of this study was to investigate morphology and reproductive health in túngara frogs collected from reference, suburban and agricultural sites utilising non-destructive methods. Despite the lack of chemicals detection in tadpole tissue, we found that morphology, male secondary sexual characteristics and breeding success were negatively impacted in frogs from agricultural compared to reference site types. Egg number (2.5-fold lower) and hatching success (2.77-fold lower) were the most severely impacted endpoints, with the reduced egg number occurring independently of the smaller female size which was also observed in frogs collected from agricultural sites (2.16-fold lower). Differences observed between reference and agricultural populations were unlikely to be an artefact of sampling effort since data collected from the different site characterisations had similar temporal (sampled during the same months) and numerical (number of sampled frogs) characteristics (Table 2). Since we did not age sampled frogs as this is an invasive process that causes harm and was inconsistent with aims of this work, we cannot rule out that different age of individuals contributed to effects observed. However, túngara frogs do not normally live for longer than 1 year (Ryan, 2010), and therefore, similar demographics between populations are expected. To the authors’ knowledge, this is the first time that male secondary sexual characteristics, egg number, hatching success or fertility in frogs collected from different types of sites have been reported in a tropical amphibian species. The methods outlined here have wide applicability since they are technically simple and cheap, and, importantly, have applicability for use in threatened/declining species since they are non-destructive.

Reduced hatching success has been demonstrated in several temperate species, for example in southern toads (*Anaxyrus terrestris*) from coal combustion sites in the USA (Metts et al. 2013) and in common toads (*Bufo bufo*) from an agricultural site in the UK (Orton and Routledge 2011). However, in contrast to our study, hatching success was reported as the proportion of a pre-determined number of collected eggs that successfully hatched, rather than the total reproductive output from a pair of amplecting frogs; so, our results are not directly comparable. Reports of total egg number in temperate species collected from polluted versus unpolluted environments are much more scarce and to the authors’ knowledge no differences have been reported to date (*Anaxyrus terrestris*, Canada: Metts et al. 2013; *Bufo bufo*, Hungary: Bókony et al. 2018; *Bufo raddei*, China: Zhang et al. 2018). With just one known publication that has recorded egg number in agricultural versus reference sites (Bókony et al. 2018), comparisons with the present study are difficult. Furthermore, in that study the difference between the females pre-spawning and post-spawning body mass was used to estimate egg number (rather than counting the number of eggs) so the results are not directly comparable to those presented here. Overall, much more research is needed on egg number in wild amphibians inhabiting a range of different environments to be able to make conclusions regarding the effects of habitat degradation and/or pollution on female reproductive health.

Hatching success was highly correlated with egg number (egg number versus number hatched tadpoles *R*^2^ values: reference = 0.97; suburban = 0.99; agricultural = 0.91), strongly indicating that reduced oviposition likely accounted for the reduced hatching success, rather than reduced fertilisation. Albeit significantly reduced in frogs collected from agricultural sites, fertilisation success in all groups was high (reference and suburban = 99%, agricultural = 97%), further indicating that impacts on male reproductive fitness may be comparatively small. Females are classically considered to be the limiting sex in populations, since they require higher levels of investment to produce gametes compared to males (Bateman 1948). It is perhaps surprising, therefore, that egg number has not been analysed more often in studies investigating reproductive health in wild amphibians. Particularly, since this endpoint is commonly used to investigate the reproductive health of wild fish (e.g. Benejam et al. 2010) and the methods for both types of organisms are similar. It was also surprising that egg number was lower in frogs collected from agricultural sites independently from female weight, since these endpoints are normally assumed to be highly correlated. This lack of correlation may be an artefact of the study design, as weights used for correlations were post-breeding values (it was not possible to record pre-spawning female weight without disrupting breeding). On the other hand, the lack of correlation between female weight and fecundity has previously been reported for another foam building species in the Leptodactylidae family (*Leptodactylus fuscusa*: Prado and Haddad 2005), providing tentative evidence that for foam building species, female size may not be an important determinator for egg number; especially as this correlation was observed for the other 6 species investigated in that study (Prado and Haddad 2005).

In addition to effects on breeding success, body condition of male and female frogs collected from agricultural sites were smaller compared to those collected from reference sites (fold-difference: female − 1.3/male − 1.16). For both male and females, the recorded SVL of frogs collected from the reference and suburban sites were within the range of those reported previously from forested areas (e.g. Ryan 1983), suggesting that frogs collected from the agricultural sites were undersized. Similarly to our results, in other tropical amphibians, common frogs (*Fejervarya limnocharis*) from agricultural sites in India (Hegde and Krishnamurthy 2014) and Thailand (Thammachoti et al. 2012) were reported to have a lower condition factor than frogs from a reference environment. By contrast, body size did not differ in the temperate species *Bufo raddei* (China: Zhang et al. 2018) or *Anaxyrus terrestris* (USA: Metts et al. 2013) in heavy metal contaminated versus reference sites, whereas natterjack toads (*Epidalea calamita*) from agricultural sites were larger than those from reference sites (Zamora-Camacho and Comas 2017). It is difficult to hypothesise regarding this apparent difference in size and/or condition between tropical and temperate species due to the limited data set, large range of different landscape types investigated and large geographic area across these studies. Male secondary sexual characteristics were also smaller in frogs from agricultural sites, and for forelimb width, this occurred independently from male weight. To date, we could find no reports of measuring these features in tropical species, however, forelimb width is controlled by androgens (Dorlochter et al. 1994) and has been shown to be reduced upon laboratory exposure to an anti-androgenic herbicide (linuron: Orton et al. 2018), and therefore, the smaller forelimb width in these frogs could possibly have been due to the presence of endocrine disrupting pollutants (for review see Orton and Tyler 2015). However, in the absence of detection of any suspected endocrine disrupting contaminants in tadpole samples, it is unknown if this explanation is likely or not.

We cannot assign observed effects to chemicals exposure, since we did not detect any of the selected chemicals in tadpole tissue. We could find no examples in the literature of chemicals being analysed in tadpoles for comparison; however, studies in adult frogs have reported relatively low detection rates of chemicals (17 out of 98: Smalling et al. 2015; 4 out of 98: Swanson et al. 2018), perhaps due to biotransformation. There is evidence that some of the pesticides we measured (paraquat, cypermethrin, glyphosate/aminomethylphosphonic acid (AMPA), profenofos) have been used in the area of Trinidad where our field sites were located (Hroudova 2012), so we may expect these chemicals to be detected. For glyphosate/AMPA, it has previously been reported that simultaneous analysis of water and frog tissue resulted in detection in the water sample only (Smalling et al. 2015); therefore, at least for these chemicals absence of detection may not accurately represent absence from the water body. Chemicals may also have been present at levels that were below the detection limit of the instruments (our detection limits were relatively high (10–50 µg/kg versus, e.g. 0.5–4.2 µg/kg: Smalling et al. 2015; Swanson et al. 2018)) and it is well known that the combined effects of low concentrations of chemical mixtures elicit effects on biota, including in amphibians (Relyea 2004; Hayes et al. 2006). However, in the absence of detected chemicals this is a hypothesis only. Beyond chemical contamination, agricultural environments are likely to have larger temperature fluctuations due to shallower pools and less shading, altered abiotic factors such as pH, dissolved oxygen and total dissolved solids, reduction in food availability and/or alterations to community dynamics (Mann et al. 2009) and interactions between environmental stressors are known to negatively impact amphibian health (Relyea 2003). Finally, since our morphological data derived from adult frogs, a temporal mismatch occurred between the biological collected from adult frogs and the tadpoles collected for chemical analysis. Therefore, it is not known if the sampled adult frogs experienced a distinct early life exposure (i.e. the previous year) compared to the sampled tadpoles (i.e. due to low site fidelity or changes in sites over time).

In conclusion, we observed significant differences between frogs collected in agricultural and reference sites with respect to morphological and reproductive endpoints, the first time that this has been reported in a tropical species. Since tropical amphibians are under threat on a global scale, this finding has significance for conservation of amphibians as species producing fewer offspring over a given time span are fundamentally at increased risk of decline (Owens and Bennett 2000). Despite evidence linking reduced reproduction and population decline in other species associated with freshwater environments (piscivorous birds, freshwater fish and alligators: see Bernanke and Köhler 2009), this evidence is currently lacking for amphibians. The methods outlined here have potential to be utilised to investigate these linkages as they are simple, cheap, informative and in the case of reproductive success, can be directly linked to population stability. Finally, tropical species may be well suited to these types of analyses since the assessment of breeding success is relatively undemanding due to the lower time required for hatching in many species compared to temperate species (< 72 h vs. 10–20 days: Duellman and Trueb 1994), resulting in reduced time and cost requirements.

## Supplementary Information

Below is the link to the electronic supplementary material.

Supplementary file1 (PDF 1666 kb)

## Data Availability

The datasets used and/or analysed during the current study are available from the corresponding author on reasonable request.
