# Supplementary material for: Non-destructive methods to assess health of wild tropical frogs (túngara frogs: Engystomops pustulosus) in Trinidad reveal negative impacts of agricultural land
Source: Environ Sci Pollut Res Int. 2022 Apr 24;29(26):40262–72. doi: 10.1007/s11356-022-20105-4 (PMC9119901; doi:10.1007/s11356-022-20105-4)
Supplement: Supplementary file 1 — Supplementary file1 (PDF 1666 kb) [file 11356_2022_20105_MOESM1_ESM.pdf]

Supplementary Material

Non-destructive methods to assess health of wild tropical frogs (túngara frogs: Engystomops pustulosus) in Trinidad reveal negative impacts of agricultural land

Frances Orton[a]*, Stephanie Mangan[b], Laura Newton[a] & Alexis Marianes[c]

* Corresponding/Senior author

a: School of Health and Life Sciences, University of the West of Scotland, Paisley, Scotland PA1 2BB b: School of Science, University of Waikato, Hamilton, New Zealand, 3216

c: Sustainable Innovation Initiatives, 9735 SW 166 Terr, Miami, FL 33157, USA

Table S1. Co-ordinates of sampling sites in Trinidad

Year	Site Name	Code	GPS Co-ordinates	Decimal co-ordinates
**2016**	Brasso Seco	BS	N10°30.155, W61°26.958	10.502583, -61.449300
**2016**	Cumuto Road	CR	N10°44.586, W61°18.569	10.743100, -61.309483
**2016**	Aripo Savannah	AS	N10°36.913, W61°12.228	10.615217, -61.203800
**2016**	Caura 1	C1	N10° 41.511, W61° 21.557	10.691850, -61.359283
**2016**	Lopinot 2	L2	N10° 40.046, W61° 19.751	10.667433, -61.329183
**2016**	Lopinot 3	L3	N10° 39.475, W61° 19.647	10.657917, -61.327450
**2016**	Lopinot 4	L4	N10° 39.475, W61° 19.574	10.657917, -61.326233
**2016**	Arena Forest	AF	N10° 35.208, W61° 13.884	10.586800, -61.231400
**2016**	Caura 2	C2	N10° 41.910, W61° 21.552	10.698500, -61.359200
**2016/18**	Lopinot 1	L1	N10°41.386, W61°19.394	10.689767, -61.323233
**2018**	Lopinot 5	L5	N10°41.406, W61°19.538	10.689777, -61.323400
**2018**	Caroni Fields	CF	N10°35.471, W61°22.820	10.591183, -61.380333
**2018**	Sant Cruz 1	SC1	N10°43.149, W61°27.526	10.719150, -61.458767
**2018**	Santa Cruz 2	SC2	N10°43.942, W61°28.180	10.732367, -61.469667
**2018**	Santa Cruz 3	SC3	N10°44.223, W61°28.323	10.737050, -61.472050

Figure S1. Google maps of selected sampling sites in Trinidad

Reference Sites

Cumuto Road (2016)

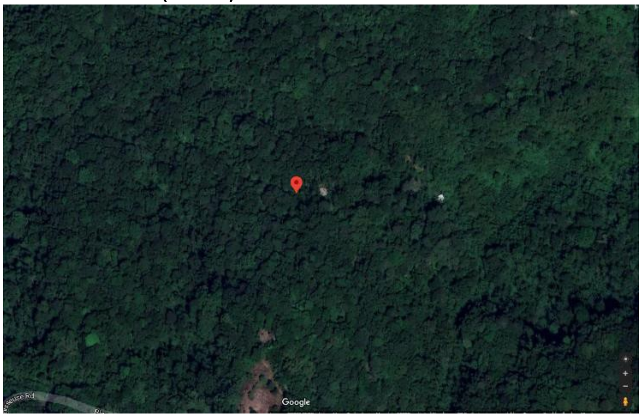

Caura 1 (2016)

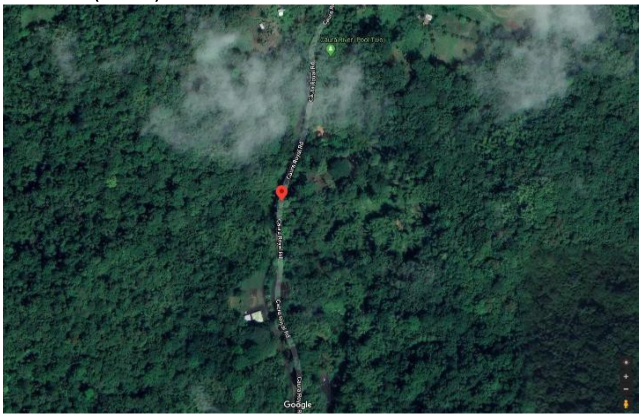

Arena Building (2016)

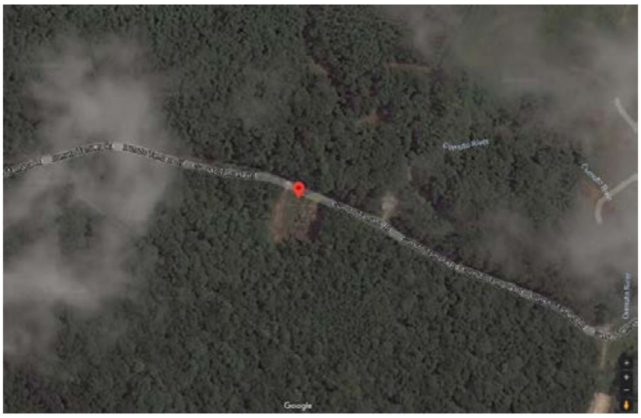

Lopinot 2 (2016)

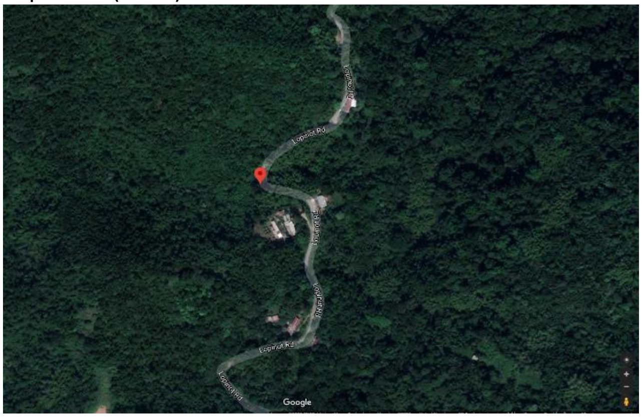

Santa Cruz 1 (2018)

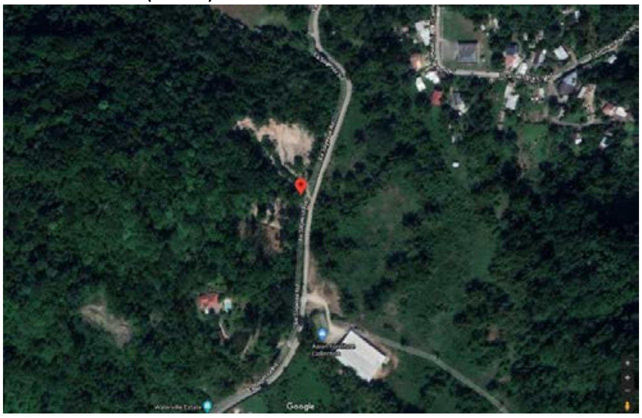

Suburban Sites

Lopinot 3 (2016)

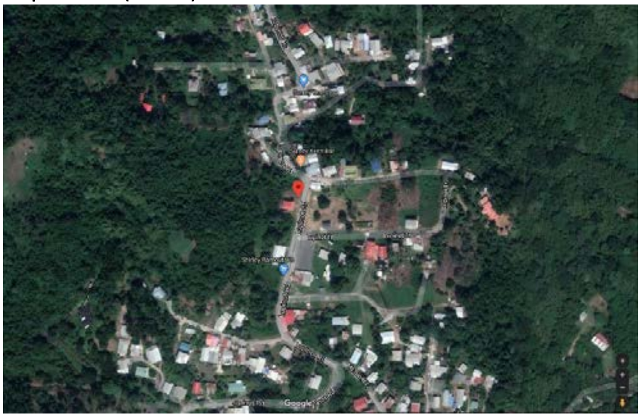

Lopinot 4 (2016)

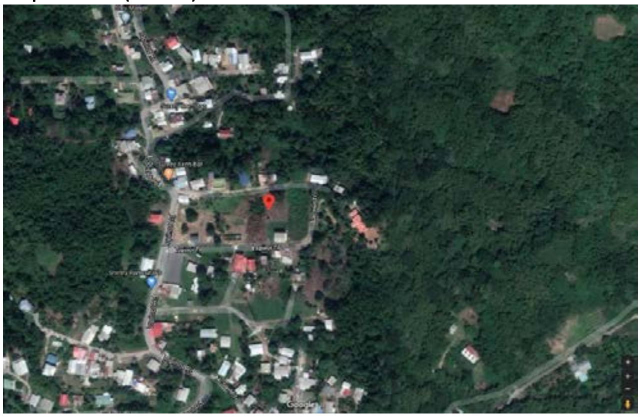

Lopinot 1 (2016/2018)

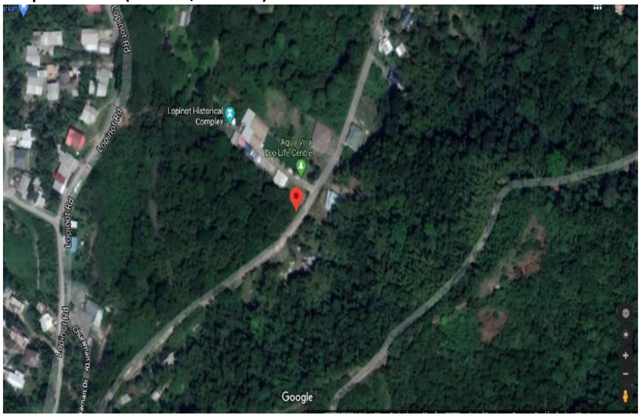

Lopinot 5 (2018)

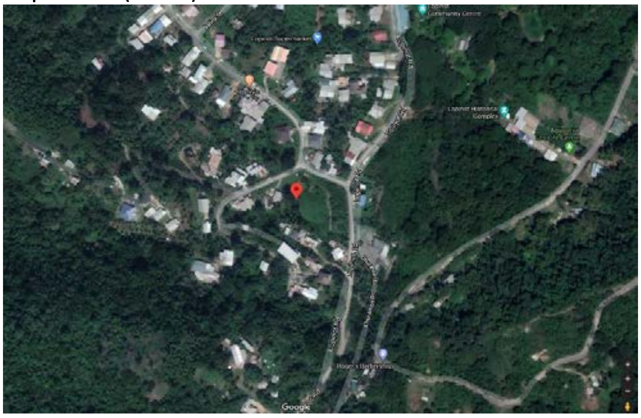

Santa Cruz 2 (2018)

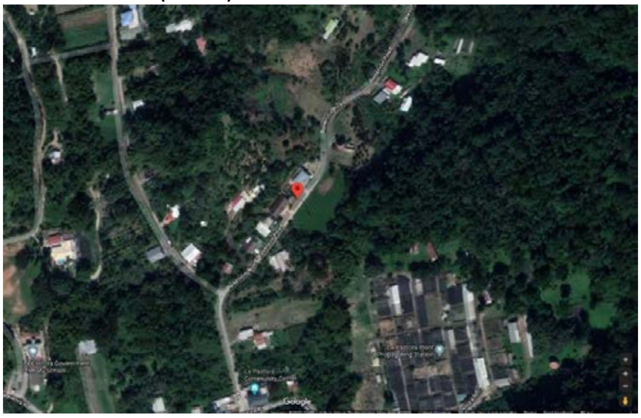

Santa Cruz 3 (2018)

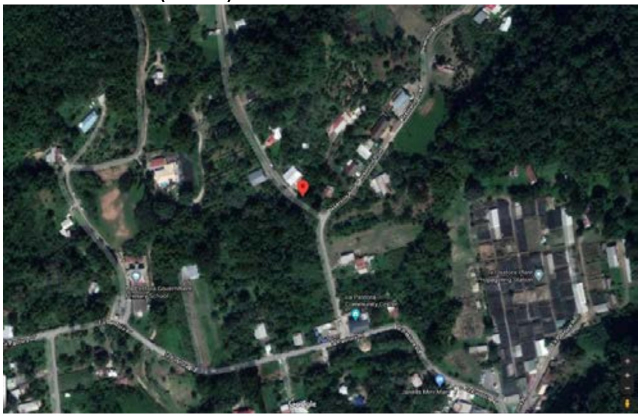

Agricultural Sites

Brasso Seco (2016)

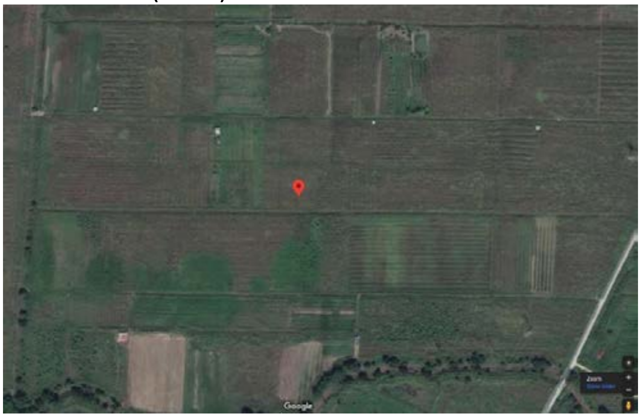

Aripo savannah (2016)

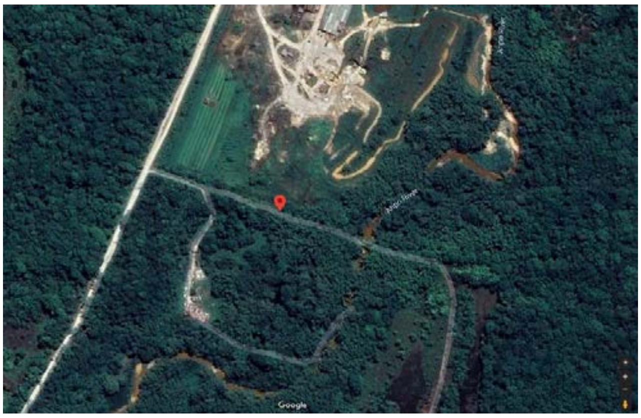

Caura 2 (2016)

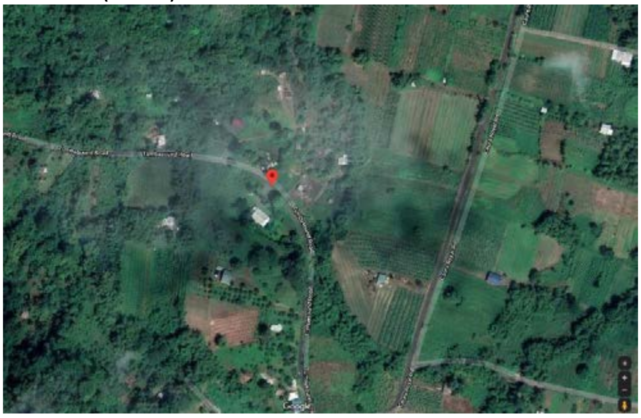

Caroni Fields (2018)

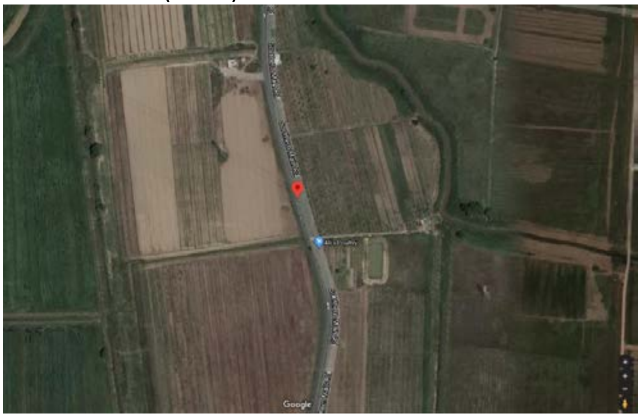

Text S1. Description of forelimb width and nuptial pad measurements

Forelimb width (2016 and 2018) and nuptial pad length (2018) were measured from photographs. These measurements could not be obtained for all frogs, as for some photographs, the forelimb was hidden under the body and/or the photograph quality was not sufficient (ie, blurry). For the photographs from 2016, fingernail width/length of a known empirical width/length were used as a scale and for the photographs from 2018, a ruler was used as a scale. All measurements were recorded twice and the mean, standard deviation and covariance of variation (COV) were calculated. If the COV was >5%, a third measurement was carried out and the two results found to be closest together were used to calculate the final mean.

Measurement Process

Select frog photo and open with adobe Photoshop

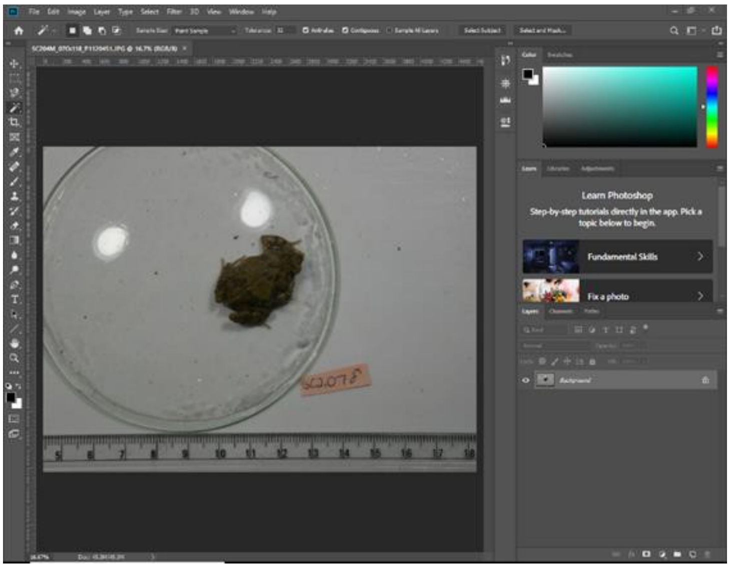

Enlarge photo as far as possible on the scale being used, e.g. the ruler in the photo below

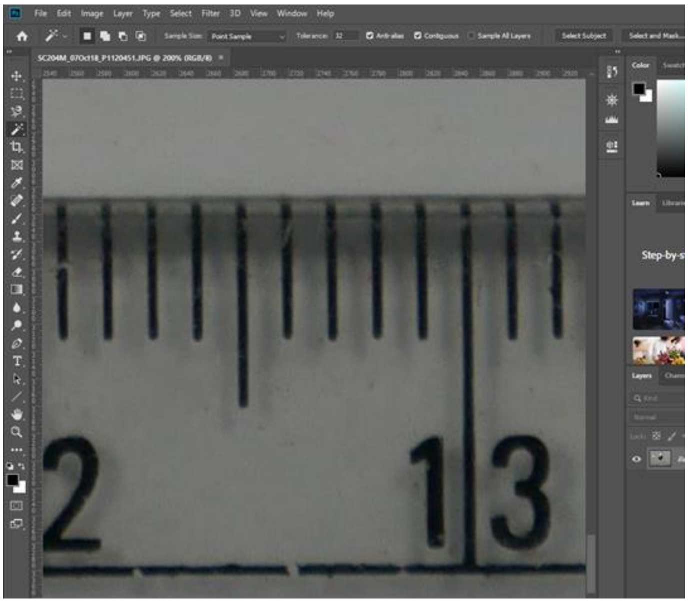

Set the scale by selecting Image – Analysis – Set Measurement Scale – Custom. A dialogue box 'Measurement Scale' then appears

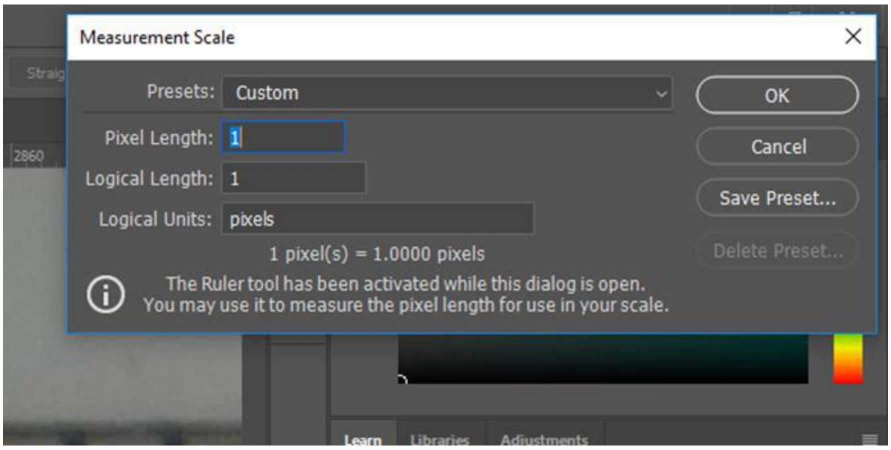

Select the Ruler tool from the options on the left and drag across the ruler ensuring the same area is standardised for every photo (left edge of mm mark)

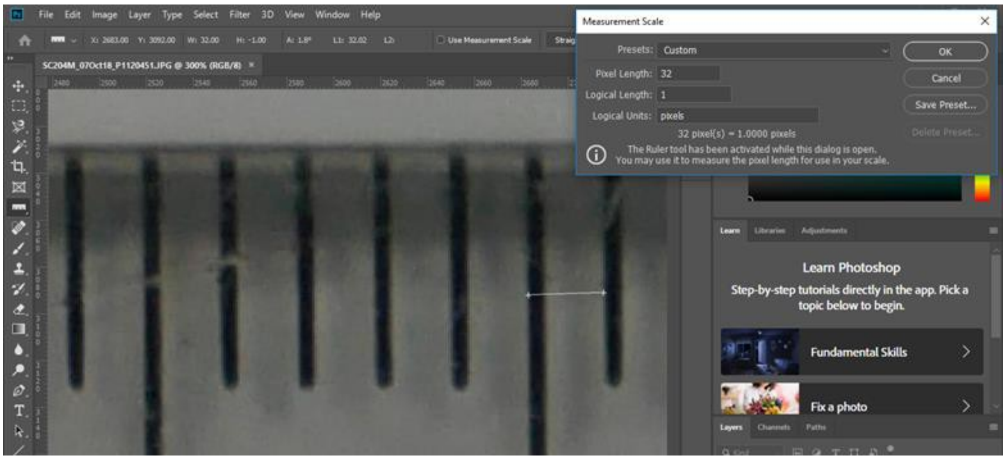

Once selected, the pixel length will appear, which is used to standardise measurements to 1mm ('X' pixels = 1mm)

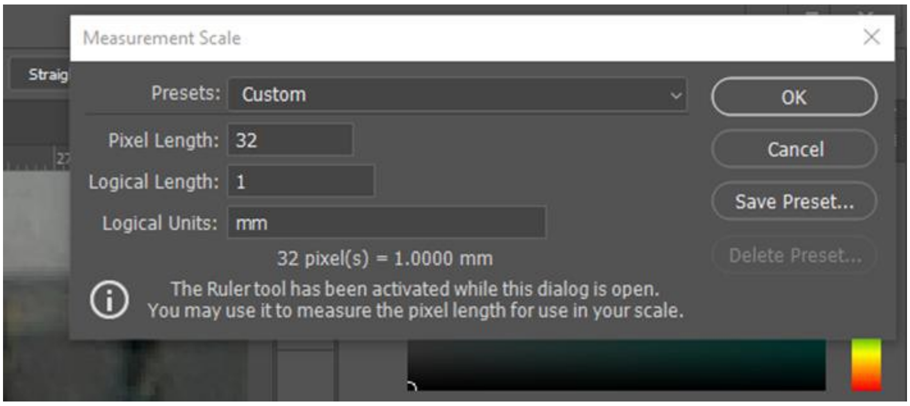

Select the 'Line tool' from options on the left – for the forelimb, drag across ensuring the line is always at a right angle to the length of the arm; for the nuptial pad length – drag the line along the length of the nuptial pad. Hold the line across the arm and without clicking off, take note of the 'Forearm Pixel Number' which is the 'L' value that appears in the box. To calculate the forelimb width/nuptial pad length the following calculation was used: forelimb pixel number/scale pixel number) * scale length.

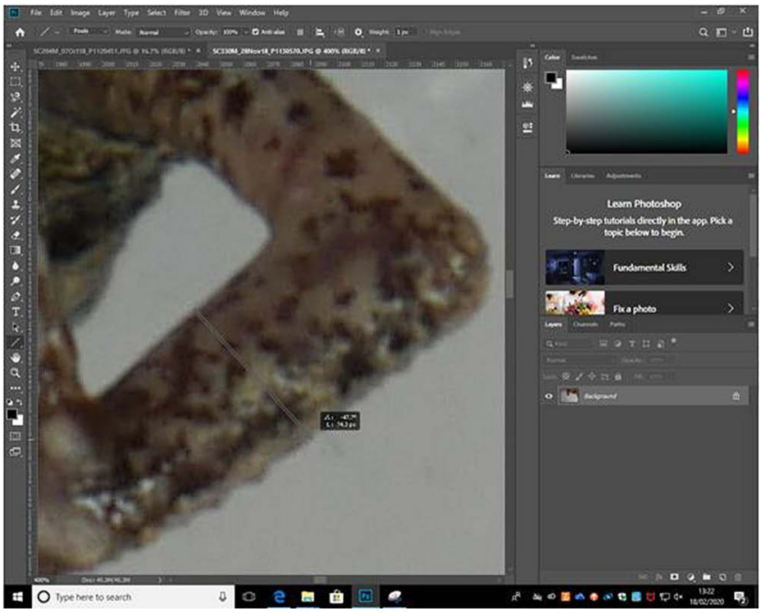
File Edit Image Layer Type Select Filter 3D View Window Help
Learn Photoshop
Step-by-step tutorials directly in the app. Pick a topic below to begin.
Fundamental Skills
Fix a photo
Type here to search

Text S2. Methods used for analysing chemicals in tadpole samples

Tadpole samples were homogenised with dry ice. For GC/MS-MS (Table S1), QuECHERS method was used: 10 g of sample, add 10 mL of acetonitrile, shake 2 min in vortex, add 4 g of magnesium sulfate, 1 g of sodium chloride, 1 g of sodium citrate dehydrate and 0.5 g of sodium citrate sesquihydrate. Then shake 2 min in vortex and centrifuge 6 min at 3500 rpm. 1 µl of extract was injected into the GC-MS. For UPLC/MS-MS (Table S2), 100 µl of extract was diluted with 900 µl water:methanol (80:20) , and 1µl was injected into the UPLC-MS. For analysis of polar compounds by UPLC/MS-MS (Table S2, identified by an asterisk), 10 g of sample was extracted with 25 mL of methanol (1% formic acid), shaken with a vortex for 4 min and centrifuged for 6 min at 3500 rpm. For glyphosate/glyphonisate, 3 µl were injected and for the other polar compounds, 5 µl was injected.

MÉTHOD OF ANÁLYSIS OF MULTIPLE PESTICIDE RESIDUES IN TADPOLES

Identification

To identify of pesticides, we use a standard deviation of retention time of these compounds between the samples and standard lower than 0,1 min.

Determination of pesticides

The procedure of extraction is based on modified QuEChERS. An aliquot of sample is analyzed by GC-MS/MS and UPLC-MS/MS. The calibration is performed on matrix with internal standard.

Equipment

For the chromatographic determination, a gas chromatograph and a liquid chromatograp of high resolution with detector of mass spectrometry (triple quadrupole) is used. As for the operational conditions they are detailed below.

Materials

- Column 2 x 15 m x 0.25 mm d.i., 0.25 µm. Factor Four VF-5 MS
- Column Waters Acquity UPLC BEH C18 (100 mm x 2.1 mm d.i., 1.7 µm)
- Balance of 0,01 mg of resolution
- Balance of 0,1 mg of resolution
- Automatic micropippetes 10-100 µL
- Automatic micropippetes 100-1000 µL
- Volumetric flask of 2 mL and 50 mL
- Vials of chromatography
- Evaporator
- Centrifuge
- Plastic tube of 15 mL and 50 mL
- Mixer
- Generator of Nitrogen
- Agitator
- Gas Helium, quality 5.0
- Gas Argon, quality 5.0
- Nitrogen extrapure
- Ethyl acetate
- Acetonitrile

- Standard of pesticide
- Sorbent of clean-up (C18, PSA, GCB)
- GC chromatography 7000C Agilent
- LC Agilent Model 1290 and MS Sciex® Model 5500

Operational conditions in GC

- Column: 2 Column of Agilent HP- 5 15mx250 μm
- Inyection temperature: solvent vent (3 mL/min, purge)
- Carrier Gas: Helio
- Program of temperature: 70ºC during 0,2 min, then 300 ºC the reason of 210ºC/min.
- Pressure/Flow in Column nº1: de 4 mL/min
- Pressure/Flow in Column nº2: de 4,8 mL/min
- Injection volume: 1 μl
- Source temperature: 280 ºC
- Transfer line temperature: 280ºC

Operational conditions in LC

- Column Waters Acquity UPLC BEH C18 (100 mm x 2.1 mm d.i., 1.7 μm)
- Temperature column: 30ºC
- Mobile phase: 5 mM ammonium formiate: MeOH
- Curtain Gas (CUR) (V): 25
- Collision Gas (CAD) (V): 9
- Ion Spray Voltage (IS)(V): 5000
- Injection volume: 5 μl
- Source temperature: 400 ºC

Validation-Quality parameters

The method is evaluated in terms of linearity, repeatability, reproducibility and accuracy. The quality parameters are detailed below

Compound	Linearity		RSD (%)[a]	RSD (%)[b]	Reproducibility		Accuracy	
	Regression coeficient (R^2)	Deviation			(%)[a]	(%)[b]	(%)[a]	(%)[b]
**Pesticide**	1.00	<20%	<20	<20	<20	<20	<50	<40

[a] calculated in limit of quantification; [b] five time limit of quantification

References

[1] Guidance document on analytical quality control and method validation procedures for pesticide residues and analysis in food and feed. Nº SANTE/12682/2019

Table S2. Chemicals analysed in tadpole samples (values in mg/kg) by GC/MS-MS

2-phenylphenol	< 0.010	Esfenvalerate+Fenvalerate	< 0.010	Profenofos	< 0.010
Aclonifen	< 0.010	Ethiofencarb	< 0.010	Propachlor	< 0.010
Acrinathrin	< 0.010	Ethion	< 0.010	Propargite	< 0.010
Azoxystrobin	< 0.010	Ethoprofos	< 0.010	Propiconazole	< 0.010
Benalaxyl	< 0.010	Etridiazol	< 0.020	Propoxur	< 0.010
Biphenyl	< 0.010	Etrimfos	< 0.010	Prothiophos	< 0.010
Bifenox	< 0.010	Famoxadone	< 0.010	Pyrazofos	< 0.010
Bifenthrin	< 0.010	Fenamifos	< 0.010	Pyridaben	< 0.010
Boscalid	< 0.010	Fenarimol	< 0.010	Pyridafenthion	< 0.010
Bromacil	< 0.010	Fenitrothion	< 0.010	Pyrifenox	< 0.010
Bromophos-ethyl	< 0.010	Fenoxicarb	< 0.010	Pyrimethanil	< 0.010
Bromophos-methyl	< 0.010	Fenpropatr n	< 0.010	Pyriproxifen	< 0.010
Bromopropylate	< 0.010	Fenthion	< 0.010	Quinalphos	< 0.010
Bupirimate	< 0.010	Fenthoate	< 0.010	Quinomethionate	< 0.010
Buprofezin	< 0.010	Fipronil	< 0.010	Quintozene	< 0.010
Butralin	< 0.010	Flucitrinato	< 0.010	S421	< 0.020
Cadusafos	< 0.010	Fludioxonil	< 0.010	Silafluofen	< 0.010
Captan	< 0.010	F olpet	< 0.010	Sulphur	< 0.010
Carbophenothion	< 0.010	Fonofos	< 0.010	Tau-fluvalinate	< 0.010
Chlordane	< 0.010	Formotion	< 0.010	Tebuconazole	< 0.010
Chlorfenapyr	< 0.010	Fosalon	< 0.010	Tecnazene	< 0.010
Chlorfenson	< 0.010	Fosmet	< 0.010	Tefluthrin	< 0.010
Chlorfenvinfos	< 0.010	Furalaxil	< 0.010	Terbutrin	< 0.010
Chlormefos	< 0.010	Furatiocarb	< 0.010	Tetrachlorvinfos	< 0.010
Chlorobenside	< 0.010	Hexaconazole	< 0.010	Tetraconazole	< 0.010
Chloropropilato	< 0.010	Iprodione	< 0.010	Tetradifon	< 0.010
Chlorotalonil	< 0.010	Isocarbofos	< 0.010	Tolclofos-methyl	< 0.010
Chlorpyrifos	< 0.010	Isofenfos	< 0.010	Tolylfluanide	< 0.010
Chlorpyrifos-methyl	< 0.010	Isofenfos-methyl	< 0.010	Transfluthrin	< 0.010
Chlortal- dimethyl	< 0.010	Lamda- cyhalothrin	< 0.010	Trifluralin	< 0.010
Chlozolinate	< 0.010	Lindane	< 0.010	Vinclozolin	< 0.010
Cresoxim-methyl	< 0.010	Malathion	< 0.010		
Cyanofenphos	< 0.010	Metalaxil	< 0.010		
Cycloate	< 0.010	Metamidophos	< 0.010		
Cyfluthrin	< 0.010	Methidathion	< 0.010		
Cynidon-ethyl	< 0.010	Methoxychlor	< 0.010		
Cypermethrin	< 0.010	Mevinfos	< 0.010		
Cyproconazole	< 0.010	Myclobutanil	< 0.010		
Cyanofenphos	< 0.010	Norflurazon	< 0.010		
Deltamethrin	< 0.010	Nuarimol	< 0.010		
Diazinon	< 0.010	Oxadixyl	< 0.010		
Dichlobenil	< 0.010	Oxifluorfen	< 0.010		
Dichlofenthion	< 0.010	p.p'-DDD + o.p-DDT	< 0.010		
Dichlofluanide	< 0.010	p.p' -DDE	< 0.010		
Dichloran	< 0.010	p.p'-DDT	< 0.010		
Diclorvos	< 0.010	Parathion	< 0.010		
Dicofol	< 0.010	Parathion-methyl	< 0.010		
Dieldrin	< 0.010	Penconazole	< 0.010		
Difenoconazole	< 0.010	Pendimethalin	< 0.010		
Di metomor f	< 0.010	Permethrin	< 0.010		
Endosulfan al fa	< 0.010	Pirimiphos-ethyl	< 0.010		
Endosulfan beta	< 0.010	Pirimiphos-methyl	< 0.010		
Endosulfan sulfato	< 0.010	Procymidone	< 0.010		
Endrin	< 0.010	Prochloraz	< 0.010		

Table S3. Chemicals analysed in tadpole samples (values in mg/kg) by UPLC/MS-MS

AMPA*	< 0. 05	Espiromesifen	< 0.010	Paclobutrazol	< 0.010
Abamectin	< 0.010	Espiroxamine	< 0.010	Paraquat*	< 0.020
Acephate	< 0.010	Ethiofencarb sulfone	< 0.010	Pencicuron	< 0.010
Acetamiprid	< 0.010	Ethiofencarb sulfoxide	< 0.010	Pirimicarb	< 0.010
Aldicarb	< 0.010	Etofenprox	< 0.010	Promecarb	< 0.010
Aldicarb-sulphate	< 0.010	Etofumesato	< 0.010	Propamocarb	< 0.010
Aldicarb-sulfoxide	< 0.010	Etoxazole	< 0.010	Pymetrozine	< 0.010
Amitraz	< 0.010	Etoxyquine	< 0.010	Piperonil-butoxide	< 0.010
Azaconazole	< 0.010	Fenazaquine	< 0.010	Pyraclostrobin	< 0.010
Azadirachtin	< 0.010	Fenbuconazole	< 0.010	Quinosol	< 0.010
Azinphos-ethyl	< 0.010	Fenbutatin oxide	< 0.010	Quinoxifen	< 0.010
Azinphos-methyl	< 0.010	Fenhexamid	< 0.010	Simacine	< 0.010
Bendiocarb	< 0.010	Fenmedifan	< 0.010	Spinosad	< 0.010
Bioaletrine	< 0.010	Fenpiroximate	< 0.010	Spirodiclofen	< 0.010
Bitertanol	< 0.010	Fenpropidine	< 0.010	Tebufenocide	< 0.010
Bromuconazole	< 0.010	Fenpropimorph	< 0.010	Tebufenpirad	< 0.010
Butocarboxim	< 0.010	Fensulfothion	< 0.010	Teflubenzuron	< 0.010
Butocarboxim-sulphoxide	< 0.010	Flonicamid	< 0.010	Terbufos	< 0.010
Butoxycarboxim	< 0.010	Flubendiamide	< 0.010	Terbutaline	< 0.010
Captafol	< 0.010	Flufenoxuron	< 0.010	Thiabendazole	< 0.010
Carbaril	< 0.010	Fluquinconazole	< 0.010	Thiacloprid	< 0.010
Carbendazime	< 0.010	Fluroclorido ne	< 0.010	Thiametoxam	< 0.010
Carbofuran	< 0.010	Flusilazol	< 0.010	Thiodicarb	< 0.010
Carbofuran-3-hydroxy	< 0.010	Flutolanil	< 0.010	Thiofanate-methyl	< 0.010
Chlorantraniliprole	< 0.010	Flutriafol	< 0.010	Thiofanox	< 0.010
Chloridazon	< 0.010	Forclorfenuron	< 0.010	Thiofanox-sulfone	< 0.010
Chlormequat*	< 0.010	Formetanate	< 0.010	Thiofanox-sulfoxide	< 0.010
Clofentezine	< 0.010	Fosfamidon	< 0.010	Triadimefon	< 0.010
Clothianidin	< 0.010	Furmecyclox	< 0.010	Triadimenol	< 0.010
Coumaphos	< 0.010	Glyphosate*	< 0. 05	Triazofos	< 0.010
Cyhexatine	< 0.010	Gluphosinate*	< 0. 05	Trichlorfon	< 0.010
Cymoxanil	< 0.010	Hexitiazox	< 0.010	Tricresyl -phosphate	< 0.010
Cyprodinil	< 0.010	Himexazole	< 0.010	Trifloxystrobin	< 0.010
Cyromazine	< 0.010	Imazalil	< 0.010	Triflumizole	< 0.010
Demeton-S-methyl	< 0.010	Imidacloprid	< 0.010	Triflumuron	< 0.010
Demeton- S-methyl-iso	< 0.010	Indoxacarb	< 0.010	Vamidothion	< 0.010
Desmetrin	< 0.010	Iprovalicarb	< 0.010		
Diclobutrazole	< 0.010	Linuron	< 0.010		
Diclofluanide	< 0.010	Lufenuron	< 0.010		
Dichlorobenzamide	< 0.010	Mecarbam	< 0.010		
Dicrotofos	< 0.010	Mepanipyrim	< 0.010		
Dietofencarb	< 0.010	Mepiquat*	< 0.010		
Difenilamine	< 0.010	Metaflumizone	< 0.010		
Diflubenzuron	< 0.010	Methiocarb	< 0.010		
Dimethoate	< 0.010	Methiocarb-sulfone	< 0.010		
Diniconazole	< 0.010	Methiocarb-sulfoxide	< 0.010		
Diquat*	< 0.020	Methomyl	< 0.010		
Disulfoton	< 0.010	Methoxyfenocide	< 0.010		
Disulfoton- sulfone	< 0.010	Monocrotofos	< 0.010		
Disulfoton-sulfoxide	< 0.010	Nitempyram	< 0.010		
Diuron	< 0.010	Ofurace	< 0.010		
Dodine	< 0.010	Omethoate	< 0.010		
Emamectin- benzoate	< 0.010	Oxamyl	< 0.010		
Epoxiconazole	< 0.010	Oxidemeton- methyl	< 0.010		

* = mg/l

Table S4. Biological measurements recorded from túngara frogs collected from reference, suburban and agricultural sites.

	**Reference**			**Suburban**					**Agricultural**				
**MALES**	**2016[a]**	**2018[a]**	**Pooled[b]**	**2016[a]**	**2018[a]**	**Pooled[b]**	***P*[d]**	**Fold change**	**2016[a]**	**2018[a]**	**Pooled[b]**	***P***	**Fold change**
Weight (g)	1.2-2.5	1.7-3.2	2.3	0.8-4.7	1.2-3.8	2.3	0.48	1	0.9-2.1	0.8-2.2	1.3	***<0.0001***	-1.77 ↓
SVL (mm)	25-32	26-33	29.5	20-37	26-32	28.0	>0.99	-1.05	22-32	22-26	24.9	***<0.0001***	-1.18 ↓
CI	0.06-0.09	0.07-0.1	0.07	0.04-0.12	0.05-0.1	0.08	0.6	-1.02	0.04-0.1	0.03-0.12	0.06	***0.0001***	-1.16 ↓
FLW (mm)	1.8-3.6	2.4-3.5	2.8[c]	1.8-3.5	2.2-3.6	2.8[c]	0.93	1	1.8-3.0	1.5-2.6	2.1[c]	***<0.0001***	-1.33 ↓
NP Length (mm)	1.3-2.0	0.8-1.9	1.5[c]	0.9-2.5	0.4-2.9	1.6	0.27	+1.06	1.0-1.7	0.4-1.3	1.30[c]	***0.026***	-1.15 ↓
**FEMALES**			**Pooled[b]**			**Pooled[b]**	***P***	**Fold change**			**Pooled[b]**	***P***	**Fold change**
Weight (g)	1.3-3.9	2-3.8	2.6	1.2-3.4	2.1-4.9	3.0	***0.003***	+1.15 ↑	0.9-2.8	1-2.2	1.2	***<0.0001***	-2.16 ↓
SVL (mm)	26-35	29-38	31.4	22-38	28-38	32.2	0.12	+1.03	21-37	24-28	24.9	***<0.0001***	-1.26 ↓
CI	0.05-0.11	0.07-0.13	0.08[c]	0.06-0.12	0.08-0.13	0.09[c]	***<0.001***	+1.13 ↑	0.03-0.09	0.04-0.08	0.06[c]	***<0.0001***	-1.3 ↓
**PAIRS**			**Pooled[b]**			**Pooled[b]**	***P***	**Fold change**			**Pooled[b]**	***P***	**Fold change**
Fecundity (number)	236-838	205-699	438.5	196-845	52-664	459.0	0.91	+1.05	100-879	91-280	175.0	***<0.0001***	-2.5 ↓
Hatching (number)	234-831	3-693	430.0	196-897	27-661	455.0	>0.99	+1.06	99-852	0-268	155.5	***<0.0001***	-2.77 ↓
Fertility (%)	98-100	0.7-100	99.07	91-100	52-100	99.09	>0.99	~1	95-100	0-100	96.89	***0.009***	-1.02 ↓

[a] Range of values, [b] Median values, [c] Mean value (condition index (CI)/forelimb width (FLW) data normally distributed). [d] *p* value refers to comparison between reference and suburban/agricultural sites with multiplicity adjusted *p* values (normally distributed data: Bonferroni; not-normally distributed data: Dunn's) . Abbreviations: SVL = snout-vent length; CI = condition index, FLW = forelimb width

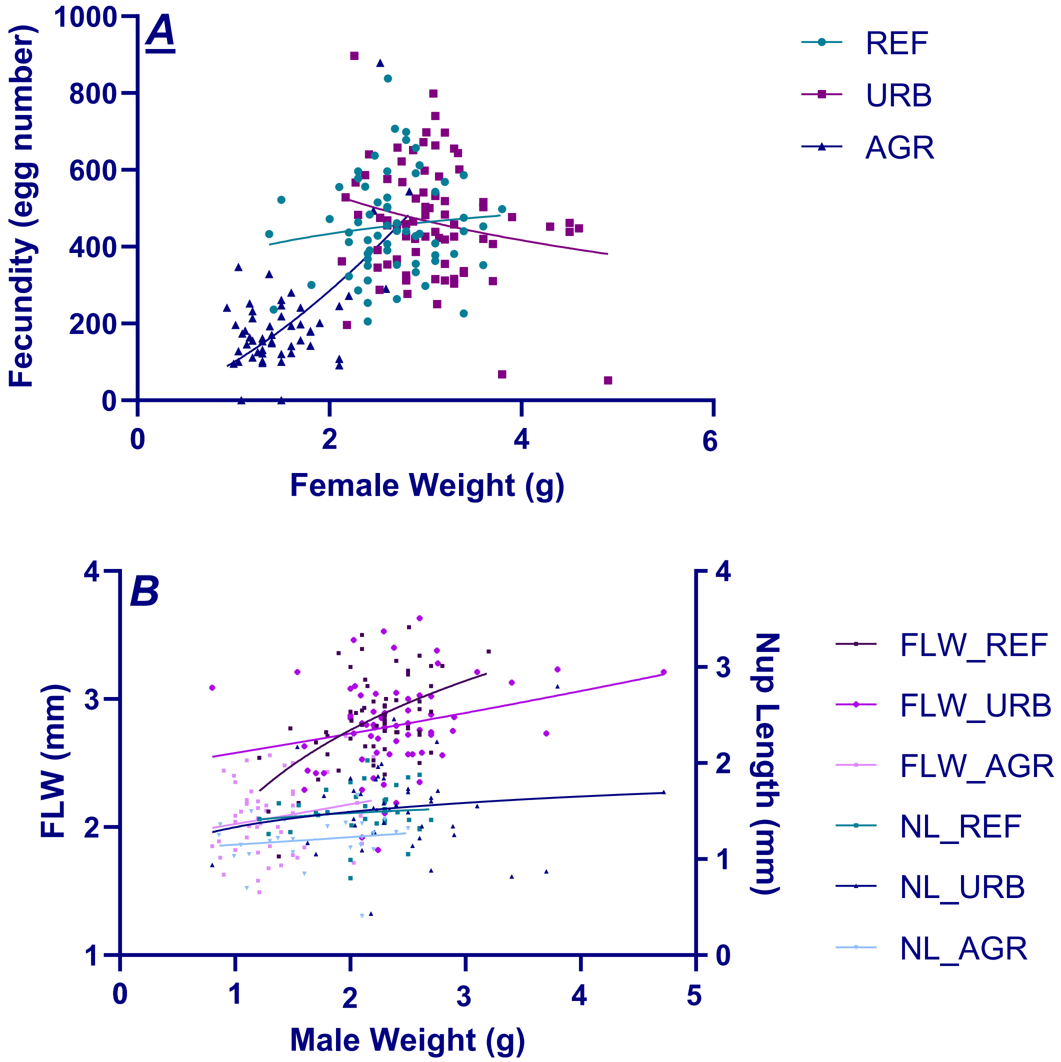

Figure S2. Correlations between female weight with fecundity (A: REF $R^2 = 0.01$; URB $R^2 = 0.04$; AGR $R^2 = 0.34$) and male weight with forelimb width (FLW: B: REF $R^2 = 0.35$; URB $R_2 = 0.06$; AGR $R_2 = 0.04$) or nuptial pad (Nup) length (B: REF $R_2 = 0.01$; URB $R_2 = 0.01$; AGR $R_2 = 0.02$) in tungara frogs collected from reference (REF), agricultural (AGR) and suburban (URB) sites.
